# Genetic Variants in Preeclampsia During Pregnancy: A Hospital-Based Case–Control Study

**DOI:** 10.3390/jcm14113850

**Published:** 2025-05-30

**Authors:** Tatyana Slobodchikova, Dana Tayzhanova, Zhanna Amirbekova, Dmitriy Vazenmiller, Ramil Mustafin, Marina Izmailovich

**Affiliations:** 1 Department of Obstetrics, Gynecology and Perinatology, Karaganda Medical University, Karaganda 100000, Kazakhstan; slobodchikova@kgmu.kz (T.S.); amirbekovaz@kgmu.kz (Z.A.); vazenmiller@qmu.kz (D.V.); 2 Department of Internal Diseases, Karaganda Medical University, Karaganda 100000, Kazakhstan; tayzhanova@qmu.kz; 3 EcoLife Clinic, Astana 010000, Kazakhstan; r.mustafin@ecolife.kz

**Keywords:** preeclampsia, genetic polymorphisms, hypertensive complications, genotype–phenotype association, reproductive health risks

## Abstract

**Background/Objectives:** Preeclampsia is a multifactorial disorder with a possible genetic component. While numerous studies have explored genetic susceptibility, validation remains inconsistent. The aim was to assess the association between hypertension-related polymorphisms and preeclampsia risk. **Methods**: A case–control study was conducted in Karaganda, Kazakhstan (n = 95). Sixty SNPs were genotyped using the QuantStudio™ 12K Flex system. Genotype–phenotype associations were evaluated using five inheritance models and statistical analysis in R. **Results**: Significant associations were found for rs2516839 (C/T: OR = 5.28; 95% CI: 1.53–18.15), rs17672135 (T/T: OR = 3.48; CI: 1.05–11.5), and rs10757278 (A/G: OR = 0.3; CI: 0.11–0.83). However, wide confidence intervals suggest potential limitations in sample size and generalizability. **Conclusions**: While these polymorphisms show promise as genetic markers of preeclampsia risk, their clinical application requires further validation in larger, multi-ethnic cohorts.

## 1. Introduction

Preeclampsia is a pregnancy-related complication and a multi-organ dysfunction syndrome that affects 3–5% of pregnancies occurring after 20 weeks of gestation, during childbirth, or in the postpartum period. It is primarily characterized by hypertension (≥140/90 mmHg) and proteinuria (≥0.3 g per 24 h of urine collection). Additional clinical manifestations may include headaches, visual disturbances, abdominal pain, and, in some cases, fluid retention. This condition is a leading cause of maternal mortality and perinatal losses and accounts for nearly one-third of preterm births, making it a significant concern in obstetric care [1,2,3,4].

Preeclampsia is a condition unique to humans, making it difficult to study using animal models. In severe cases, it can lead to hypertension, renal insufficiency, and multi-organ dysfunction, including hepatic impairment and HELLP syndrome, significantly increasing the risk of long-term health complications for the mother. As a progressive disorder, preeclampsia manifests clinically only after its pathological processes are already underway, with the onset of symptoms marking its transition to a clinically apparent stage [5,6,7].

Currently, there is no effective pathogenetic therapy for preeclampsia, and delivery remains the only definitive treatment. Several risk factors have been identified that increase susceptibility to preeclampsia, including chronic arterial hypertension, chronic kidney disease, a personal or family history of preeclampsia, and certain reproductive factors. Women at higher risk include those experiencing their first pregnancies at young ages or over 35 years old or those with new partners, an intergenital interval exceeding 10 years, or pregnancies conceived through assisted reproductive technologies. Additionally, obesity and African–American ethnicity have been associated with an elevated risk of developing preeclampsia [8,9,10,11].

Although the exact etiology of preeclampsia remains incompletely understood, research suggests that placental dysfunction leading to impaired uteroplacental blood flow is a major contributing factor [12,13,14]. Recent studies have also highlighted the roles of altered angiogenic signaling, immune maladaptation, and endothelial dysfunction as central to the disease development [15,16]. Additionally, the familial clustering of preeclampsia cases indicates a potential genetic predisposition, with contributions from the maternal, fetal, and possibly paternal genomes [17,18,19].

Research indicates that women with first-degree relatives affected by preeclampsia have a three- to fivefold increased risk of developing the condition themselves [20,21,22]. In some families, preeclampsia appears to follow a Mendelian inheritance pattern, suggesting the involvement of rare deleterious genetic variants [23,24]. Large-scale family studies have identified associations between preeclampsia and genetic variations in ACVR2 (activin A receptor type 2A), ROCK2 (coiled-coil-containing protein kinase 2), ERAP1 (endoplasmic reticulum aminopeptidase 1), and ERAP2 (endoplasmic reticulum aminopeptidase 2) [25,26,27]. However, in most cases, the genetic basis of preeclampsia appears to be polygenic, involving multiple interacting genes rather than a single inherited mutation [28,29,30,31].

The Human Genome Project has established the universality of the genetic code; however, genes encode proteins whose functions and expressions can be influenced by various physiological and environmental conditions. Pregnancy represents a unique physiological state, particularly in cases of multifetal gestation or conception via assisted reproductive technologies, complicating the identification of the universal genetic polymorphisms associated with preeclampsia. This study aimed to assess the probability of preeclampsia occurrence based on genetic polymorphisms linked to hypertension risk. Our findings may contribute to the advancement of personalized medicine, enabling an individualized approach to pregnancy management and the optimization of delivery timing. Furthermore, this research may facilitate the development of a genetic health passport for women of reproductive age or those planning pregnancies, providing valuable insights for risk assessment and preventive strategies.

## 2. Methods

This hospital-based case–control study was conducted at the Regional Perinatal Center in Karaganda (Kazakhstan), which provides advanced care for high-risk pregnancies. This study was carried out following protocol No. 20, approved by the bioethics committee of NPJSC “Karaganda Medical University” on 17 June 2019. Participant recruitment took place from July 2019 to December 2020. All participants completed a standardized questionnaire (in Russian or Kazakh) and underwent routine clinical and laboratory assessments during hospitalization.

The eligible participants were pregnant women aged 18–39 years, each with a BMI of 22–28 kg/m^2^, a parity of one to three, and spontaneous conception. All resided in the Karaganda city or region and were enrolled in antenatal care between the 10th and 12th gestational weeks. The baseline blood pressure ranged from 110/70 to 120/80 mmHg. Mild chronic conditions such as pyelonephritis, anemia, compensated varicose veins, or cholecystitis were permitted.

The participants were stratified into two groups. The study group consisted of high-risk women who received 75 mg of aspirin daily. The control group included women without risk factors and with no aspirin. The risk criteria included prior hypertensive pregnancy, chronic kidney or cardiovascular disease, nulliparity, maternal age of ≥ 40, an interpregnancy interval of ≥ 10 years, a BMI of ≥ 35 kg/m^2^, or multiple gestation.

The median participant age was 28.7 years (IQR: 25.4–32.1). Nulliparity was present in 38.5% of the women, and 13% had histories of chronic hypertension. The baseline characteristics between the groups were comparable.

The exclusion criteria included a maternal age of < 18, IVF pregnancy, Rh incompatibility, severe comorbidities, infections, autoimmune disorders, and respiratory or hematologic conditions.

### 2.1. Sample Size Calculation and Power Analysis

The required sample size was determined using G*Power software. The required sample size calculation was conducted for the entire cohort, comprising the study and control groups. Based on an alpha level of 0.05, a power of 80%, and an assumed medium effect size (Cohen’s d = 0.5), the minimum required sample size was 92 participants. To account for potential dropouts, 95 participants (study: n = 45, controls: n = 50) were recruited. This allocation aimed to maintain group comparability and statistical robustness, ensuring adequate power to detect meaningful genetic associations with preeclampsia while also accounting for potential dropouts or exclusions.

### 2.2. Genotyping and Quality Control Measures

Blood samples were collected and transported to the Karaganda Medical University Research Center for real-time PCR analysis of the genetic polymorphisms associated with hypertension risk. Genotyping was conducted using the QuantStudio™ 12K Flex Real-Time PCR system (Applied Biosystems, Foster City, CA, USA). The genetic panel, consisting of 60 polymorphisms, was specifically selected based on the participants’ prior documented associations with hypertension, cardiovascular diseases, vascular dysfunction, inflammation, and endothelial dysfunction—pathophysiological processes relevant to preeclampsia. The chosen SNPs included variants located in key genes such as NOS3 (endothelial nitric oxide synthase), F5 (Leiden factor), TNFSF4 (cytokine signaling), and CDKN2B-AS1 (cell cycle regulation), which have previously been implicated in hypertensive pregnancy disorders and vascular pathology. This panel is exploratory yet informed by the existing literature, aiming to identify genetic predictors of preeclampsia and to evaluate their potential applicability in clinical risk assessment.

Reference alleles, which each correspond to the most prevalent allele in the control group, were defined based on frequency data from the 1000 Genomes Project.

### 2.3. Statistical Analysis

Statistical analysis was carried out using the R statistical software version 4.3.2 (CompareGroups version 4.6.2 and static packages version 1.0.1), with the Shapiro–Wilk test assessing data normality. Comparisons between the two groups were conducted using Student’s *t*-test for normally distributed data or the Mann–Whitney U test for non-normally distributed data. For comparisons across multiple groups, ANOVA was applied to the normally distributed data, while the Kruskal–Wallis test was used for the non-normally distributed data. The categorical variables were analyzed using the Chi-square test or Fisher’s exact test. Genotype–phenotype associations were assessed through dominant, codominant, recessive, supra-dominant, and logarithmic inheritance models. Hardy–Weinberg equilibrium (HWE) testing was performed in the control group to verify population genetics assumptions, with a significance threshold of *p* > 0.05 for all tested SNPs. A *p*-value of <0.05 was considered statistically significant.

## 3. Results

A panel of 60 single-nucleotide polymorphisms (SNPs) analyzed in this study, along with their chromosomal localization and gene associations, is presented here. The polymorphisms are located both in the coding regions of genes (TNFSF4, F5, NOS3, LPL, CDKN2B-AS1) and in regulatory non-coding areas (LINC00841, AL137026.2), allowing the assessment of their potential impact on both protein amino acid sequences and gene expression. Many of these SNPs have been previously associated with cardiovascular diseases, inflammatory processes, and vascular remodeling, highlighting their potential role in the pathogenesis of preeclampsia. Of particular interest are the SNPs within the NOS3 (endothelial nitric oxide synthase), F5 (Leiden factor), TNFSF4 (cytokine pathway), and CDKN2B-AS1 (cell cycle regulation) genes, which may play a key role in the development of vascular dysfunction and hypertension in this pathology (Table S1).

Data on the minor allele frequency (MAF) of the studied SNPs across different ethnic groups, based on the 1000 Genomes Project dataset, are presented here. The results demonstrate significant interpopulation variability, which may reflect genetic differences in predisposition to preeclampsia among ethnic groups. For example, rs599839, which is associated with lipid metabolism, is highly prevalent among East Asians (93.45%) but significantly less common among Europeans (77.83%) and Africans (17.85%). Similarly, rs3184504, involved in immune regulation, exhibits a high degree of variability, with a frequency of 98.11% in Africans compared with only 53.58% in Europeans. These differences underscore the importance of considering ethnic backgrounds when analyzing genetic risk factors for preeclampsia and planning future studies (Table S2).

**Figure 1:**
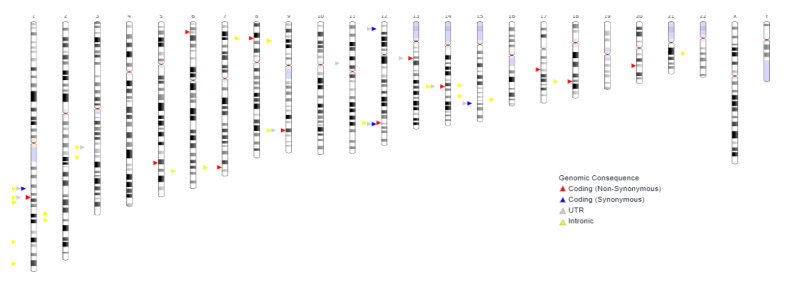
Chromosomal map showing SNPs.

A chromosomal map indicating the localization of the studied SNPs is illustrated here. This figure provides a visual representation of the distribution of the polymorphisms across the genome, covering a wide range of autosomal and sex chromosomes. Such spatial dispersion of the SNPs may reflect their contributions to various molecular pathways involved in the pathogenesis of preeclampsia, including inflammation, angiogenesis, and vascular tone regulation (Figure 1).

The primary genotyping indicators for the studied SNPs in the preeclampsia patients (GROUP = 1) and the control group (GROUP = 0) are presented, including allele frequencies, genotype distributions, and Hardy–Weinberg equilibrium (HWE) *p*-values. Notable differences between the groups suggest potential SNP involvement in preeclampsia susceptibility. rs17672135 shows a higher minor allele frequency in the preeclampsia group (A2.p = 0.056) compared to the controls (A2.p = 0.182), indicating a possible association, though the HWE remains stable. rs1799963 demonstrates significant deviations from the HWE in both groups (*p* = 0.000004 in the controls and *p* = 0.000000 in the patients), suggesting strong genetic selection or disease association. rs10757278 exhibits a higher minor allele frequency in the preeclampsia cases (A2.p = 0.446 vs. 0.561 in controls), with a marked deviation from the HWE (*p* = 0.004521), reinforcing its potential relevance. In contrast, rs268 shows no minor allele presence in the preeclampsia group (A2.p = 0), suggesting a possible protective role. These findings highlight rs17672135, rs1799963, and rs10757278 as potential genetic markers for preeclampsia, warranting further investigation (Table 1).

The statistical analysis results based on the allelic model, assessing the significance of the allele frequency differences between groups, are presented. Among the 60 studied polymorphisms, only rs17672135 exhibited statistically significant differences between the preeclampsia patients and the control group (*p* = 0.04), suggesting its potential role in disease pathogenesis. Additionally, several SNPs showed a trend toward statistical significance, including rs2516839 (*p* = 0.195), rs10757278 (*p* = 0.158), and rs2713604 (*p* = 0.054), warranting further investigation in larger cohorts. These findings indicate the possible involvement of these polymorphisms in processes related to vascular remodeling, inflammation, and endothelial dysfunction, making them promising candidates for further research (Table 2).

The genotype–phenotype association analysis results using five different inheritance models—codominant, dominant, recessive, overdominant, and log-additive—are presented here. The polymorphisms rs17672135, rs2516839, and rs10757278 exhibit significant differences across multiple inheritance models, suggesting their potential impact on preeclampsia development. Specifically, rs17672135 demonstrates significant differences in the log-additive model (*p* = 0.032), while rs10757278 shows a significant effect in the overdominant model (*p* = 0.018), which may indicate a protective role of the heterozygous state. These findings highlight the importance of a multi-level approach in analyzing the genetic predictors of preeclampsia (Table 3).

An evaluation of the association between the genotypes and phenotypes, based on the calculation of odds ratios (ORs) and 95% confidence intervals (CIs) and both with and without age adjustment, is provided here. Significant associations were observed for rs17672135, rs10757278, and rs2516839, suggesting their potential roles in disease susceptibility. rs17672135 demonstrated a significant association in the recessive model (*p* = 0.0085), with the homozygous carriers of the minor allele (T/T) showing increased risk of preeclampsia (OR = 3.48, 95% CI: 1.05–11.5). rs10757278 exhibited significant associations in the overdominant model (*p* = 0.0175), indicating a possible protective effect of the heterozygous genotype (A/G). rs2516839 showed strong associations in both the dominant and recessive models, with the T/T carriers exhibiting a significantly increased risk of preeclampsia (OR = 7.89, *p* = 0.0019) (Table 4).

## 4. Discussion

This study identified significant associations between three SNPs—rs17672135, rs2516839, and rs10757278—and the risk of preeclampsia in pregnant women. The C/T genotype of rs2516839 and the T/T genotype of rs17672135 were associated with higher odds of hypertensive complications, while the A/G genotype of rs10757278 appeared to have a protective effect. These findings suggest that genetic variability contributes to individual susceptibility to preeclampsia.

Genetic polymorphisms represent natural variations within the genome, and genetic pathologies may not manifest immediately, as their development can occur at any age and be influenced by external factors, such as pregnancy. Notably, diseases with similar clinical presentations may have distinct genetic origins, which explains why some individuals without identifiable risk factors still develop these conditions [32]. The study of polymorphisms enables the identification of high-risk groups for various diseases by examining the associations between genetic variants and disease prevalence. Prevention strategies primarily focus on minimizing external risk factors, while environmental modifications may also influence the disease process. Additionally, mutations observed in isolated cases often result from natural selection, and certain hormones can enhance mutagenesis, suggesting that IVF-induced pregnancies may carry an elevated genetic risk for certain conditions [33,34,35,36].

Previous studies [37,38] have reported an association between rs17672135 and an increased risk of cardiovascular disease, particularly coronary heart disease, without accounting for individual patient characteristics. In our study, which focused on female patients undergoing close monitoring for hypertension-related pregnancy complications, the homozygous risk allele (T) distribution demonstrated an odds ratio (OR) of 3.48 (95% confidence interval: 0.97–12.48), with a *p*-value of 0.04. These findings suggest that the T/T genotype is associated with a 3.48-fold increased risk of hypertension-related complications during pregnancy, highlighting the potential role of rs17672135 in preeclampsia susceptibility.

This variant may influence vascular remodeling and angiogenesis, potentially disrupting normal placental function and contributing to the development of maternal hypertension and proteinuria. Moreover, the location of rs17672135 in genomic regions linked to endothelial regulation suggests functional roles in nitric oxide synthesis and vascular tone. Endothelial dysfunction is a hallmark of preeclampsia and includes reduced vasodilation, increased oxidative stress, and inflammatory activation. The variant may thus impair uteroplacental blood flow, contributing to the hypertensive phenotype characteristic of the disorder [39,40].

The rs17672135 polymorphism is located in a region associated with vascular regulation and endothelial homeostasis, both of which are critical in preeclampsia pathogenesis. Endothelial dysfunction is a hallmark of preeclampsia, characterized by impaired nitric oxide (NO) production, increased oxidative stress, and pro-inflammatory signaling. This variant may influence vascular remodeling and angiogenesis, potentially disrupting normal placental function and contributing to the development of maternal hypertension and proteinuria. Given the observed association of rs17672135 with preeclampsia risk, this polymorphism may affect endothelial responses, increasing susceptibility to vascular complications during pregnancy [41].

The rs10757278 variant is located near genes involved in vascular tone regulation and blood pressure control, making it highly relevant to preeclampsia risk. This SNP has been linked to increased expression of genes associated with renin–angiotensin system (RAS) dysregulation, which plays a critical role in blood pressure regulation during pregnancy. Dysregulation of angiotensin II signaling can lead to vasoconstriction, reduced uteroplacental perfusion, and increased systemic vascular resistance, all of which are key contributors to preeclampsia development. Furthermore, rs10757278 may be involved in atherosclerosis-like changes in placental vasculature, contributing to the pro-hypertensive state observed in preeclamptic patients [42].

The rs10757278 polymorphism has been linked to increased risk of cardiovascular disease (CVD) and diabetes, with an estimated 20–30% of cardiovascular events associated with this SNP. The G risk allele is particularly correlated with myocardial infarction (MI). According to a previous study [43], individuals with the homozygous rs10757278 G/G genotype showed a significantly higher risk of multivessel coronary artery disease (CAD), with an odds ratio (OR) of 2.85 (95% CI: 1.26–6.19) for three-vessel disease and 1.95 (95% CI: 1.13–3.35) for two-vessel disease compared with those with the A/A genotype. Moreover, the G/G genotype has been associated with specific arterial involvement, including left anterior descending (LAD) artery stenosis (OR = 2.28; 95% CI: 1.27–3.52), right coronary artery (RCA) stenosis (OR = 1.26; 95% CI: 1.24–3.32), and left circumflex (LCX) artery stenosis (OR = 1.12; 95% CI: 1.04–2.26). Additionally, binary logistic regression has revealed that the rs10757278 G allele has independently increased the risk of significant coronary stenosis (OR = 1.82; 95% CI: 1.17–2.57), underscoring the potential of this SNP as a marker of CAD severity in the Saudi population.

According to a recent study, the minor G allele of rs10757278 was highly prevalent (84%) even among asymptomatic individuals. When stratified using the Framingham risk score, the frequency of the G allele increased progressively from the low- (74%) to moderate- (77%) and high-risk groups (93%). This trend was especially pronounced in males, with the G allele frequency rising from 76% in the low-risk group to 94% in the high-risk group. In contrast, this incremental pattern was not observed in females. Furthermore, the association between gender, risk status, and genotype was statistically significant (*p* < 0.001), suggesting that carriers of the rs10757278 G/G genotype, particularly men, may be at a higher latent risk of developing cardiovascular disease, even in the absence of clinical symptoms [44].

In our study of pregnant women at risk of severe preeclampsia and those with confirmed hypertensive complications, we found that the A/A genotype of rs10757278 was associated with a reduced risk of hypertensive conditions during pregnancy, with an odds ratio of 0.3 (95% CI: 0.11–0.83, *p* = 0.01). Conversely, our data suggest that the G/G genotype may increase the risk of developing hypertensive disorders, including severe preeclampsia; however, this association did not reach statistical significance (*p* > 0.05). These findings indicate a potential protective role of the A/A genotype and warrant further investigation in larger cohorts to better understand the genetic contribution of rs10757278 to preeclampsia susceptibility.

A study [45] has reported that individuals carrying the risk alleles of rs2516839 have twice the risk of sudden cardiac death compared with those with the C/C genotype, particularly among T/T genotype carriers. Another study [46] demonstrated that the risk alleles of the USF1 gene are predictive of both cardiovascular disease and severe preeclampsia development during pregnancy. Further research [47] investigated the genetic interaction between USF1 and APOA5, revealing their combined influence on lipid levels and atherosclerosis progression. A study by Wang (2013) [48] linked USF1 polymorphisms to the total area of unstable carotid plaques in ischemic stroke patients, while Niemiec (2015) [49] showed that rs2516839 modulates serum triglyceride levels in response to smoking. Additionally, Fan (2014) [50] explored the role of USF1 allelic variants in female lipoprotein metabolism and USF1 expression in atherosclerotic plaques.

In our study, we found that the C/T genotype of rs2516839 was associated with a 5.28-fold increased risk (95% CI: 1.53–18.15) of hypertensive complications during pregnancy, while the T/T genotype conferred a 2.08-fold higher risk (95% CI: 0.46–9.51, *p* = 0.02). These findings suggest that rs2516839 may influence the development of hypertensive disorders in pregnancy, warranting further investigation into its role in preeclampsia pathogenesis.

The implicated SNPs are located in genes involved in vascular tone regulation, inflammation, and endothelial function—pathways central to preeclampsia pathogenesis. For instance, NOS3 influences nitric oxide synthesis, while USF1 regulates lipid metabolism and vascular inflammation. These mechanisms are supported by prior research linking impaired angiogenesis and oxidative stress to preeclampsia development [41].

Polymorphisms contribute to genetic diversity and play a role in multifactorial diseases, which require the interaction of both hereditary and environmental factors for their manifestation [32]. In families with recurrent cases of similar pathologies, a genetic component may be involved, although chromosomal syndromes are typically observed in only one family member [51]. Preeclampsia, for instance, is largely resistant to standard therapies, with delivery being the only definitive treatment [4,52].

## 5. Limitations

This study has several limitations that should be considered when interpreting the results. Although the calculated sample size was sufficient to detect moderate genetic associations, the relatively small cohort may lack the power to identify weaker or rare variant effects. This limitation may explain the wide confidence intervals observed for several SNPs, which in turn reduces the precision and reproducibility of the effect estimates. Additionally, the genotyping was limited to a predefined panel of 60 SNPs, which may not have captured all relevant genetic variants associated with preeclampsia risk. Despite rigorous genotyping protocols and quality control measures—such as high call rate thresholds and Hardy–Weinberg equilibrium testing—technical errors or genotype misclassification cannot be entirely excluded.

Furthermore, the study population was limited to pregnant women from a single region in Kazakhstan. While this regional focus allowed for a controlled setting and reduced population heterogeneity, it also restricts the generalizability of these findings to other ethnic groups or geographic settings with different genetic backgrounds or environmental exposures. This study did not account for environmental or lifestyle factors such as diet, physical activity, stress, or exposure to environmental toxins, nor did it investigate gene-environment interactions. These unmeasured confounders could influence both genetic expression and disease manifestation. Moreover, no formal correction for multiple testing (e.g., Bonferroni or FDR) was applied, as the primary aim of this study was exploratory. While this approach allows the identification of potentially relevant associations, it also increases the risk of type I errors. Therefore, the observed associations—particularly those with marginal significance (e.g., rs17672135, *p* = 0.04)—should be interpreted with caution. Finally, the cross-sectional design of this study precludes conclusions about causal relationships. Longitudinal studies with larger, multi-ethnic cohorts and integrative analyses of clinical, genetic, and environmental data are needed to validate and extend these findings.

## 6. Conclusions

This study demonstrates that genetic polymorphisms rs17672135, rs2516839, and rs10757278 are associated with preeclampsia risk in pregnant women. The findings highlight the potential role of genetic variability in influencing susceptibility to hypertensive complications during pregnancy. However, given the limited sample size and ethnic specificity of the study population, further large-scale, multi-ethnic investigations are warranted to validate these associations and to explore their clinical applicability for personalized risk assessment and management strategies in obstetric care.

## Figures and Tables

**Table 1:** Primary genotyping indicators.

SNP	Group	Ntotal	A1	A2	A1.p	A2.p	Hom1	Het	Hom2	Hom1.p	Het.p	Hom2.p	HWE.p
rs2516839	0	44	C	T	0.65	0.35	C/C	C/T	T/T	0.5	0.3	0.2	0.118973
rs2516839	1	50	C	T	0.517	0.483	C/C	C/T	T/T	0.2	0.633	0.167	0.231098
rs17672135	0	44	T	C	0.818	0.182	T/T	C/T	C/C	0.697	0.242	0.061	0.561007
rs17672135	1	50	T	C	0.944	0.056	T/T	C/T		0.889	0.111		0.23904
rs1799963	0	44	G	A	0.935	0.065	A/A	G/G		0.065	0.935		0.000004
rs1799963	1	50	G	A	0.984	0.016	G/G	A/G		0.968	0.032		0
rs268	0	44	A	G	0.978	0.022	A/A	A/G		0.957	0.043		0.000002
rs268	1	50	A		1		A/A			1			1
rs10757278	0	44	G	A	0.439	0.561	A/A	A/G	G/G	0.303	0.515	0.182	0.929546
rs10757278	1	50	A	G	0.554	0.446	A/A	A/G	G/G	0.432	0.243	0.324	0.004521

A1/A2: major and minor alleles; Hom1/Hom2: homozygous for A1 or A2, respectively (e.g., C/C or T/T); Het: heterozygous genotype (e.g., C/T); OR: odds ratio; CI: confidence interval.

**Table 2:** Results of statistical analysis based on the allelic model (*p* < 0.1).

Rs	Reference Allele	Group	Allele_Count	Allele_ChiSq_p
rs2516839	C	0 and 1	C 39 31|T 21 29	0.195
rs17672135	T	0 and 1	T 54 68|C 12 4	0.04
rs2713604	T	0 and 1	C 26 43|T 24 17	0.054
rs10757278	A	0 and 1	A 37 41|G 29 33	0.158

**Table 3:** Table of genotype–phenotype association results (*p* values) using five-model inheritance.

RS	Codominant	Dominant	Recessive	Overdominant	Log-Additive
rs2516839	0.02099012	0.01366728	0.73850577	0.00896657	0.14975684
rs17672135	0.06454852	0.04535291	0.08246722	0.14821373	0.03228683
rs17576	0.14034989	0.05884431	0.22915966	0.50011448	0.06086446
rs688034	0.03574551	0.3213518	0.01020996	0.89354502	0.1006745
rs10757278	0.05757895	0.26176113	0.16953678	0.01808611	0.94478469

**Table 4:** Results of statistical analysis of the genotype–phenotype association.

Rs	Model Inheritance	GROUP 0	GROUP 1	OR 95 CI	*p*-Value	OR 95 CI Adj. by Age	*p*-Value Adj by Age
rs17672135	Codominant						
	C/C	2 (6.1%)	0 (0%)	1	0.10244	1	0.07282
	C/T	8 (24.2%)	4 (11.1%)	NA [0–NA]		NA [0–NA]	
	T/T	23 (69.7%)	32 (88.9%)	NA [0–NA]		NA [0–NA]	
	Dominant						
	C/C	2 (6.1%)	0 (0%)	1	0.22506	1	0.02857
	C/T-T/T	31 (93.9%)	36 (100%)	NA [0–NA]		NA [0–NA]	
	Recessive						
	C/C-C/T	10 (30.3%)	4 (11.1%)	1	0.04535	1	0.20085
	T/T	23 (69.7%)	32 (88.9%)	3.48 [0.97–12.48]		2.85 [0.54–15]	
	Overdominant						
	C/C-T/T	25 (75.8%)	32 (88.9%)	1	0.14821	1	0.63632
	C/T	8 (24.2%)	4 (11.1%)	0.39 [0.11–1.45]		0.68 [0.13–3.48]	
	Log-Additive						
	0,1,2	33 (47.8%)	36 (52.2%)	3.36 [1.04–10.91]	0.10244	3.52 [0.83–14.99]	0.07328
rs10757278	Codominant						
	A/A	10 (30.3%)	16 (43.2%)	1	0.05758	1	0.05801
	A/G	17 (51.5%)	9 (24.3%)	0.33 [0.11–1.02]		0.18 [0.04–0.9]	
	G/G	6 (18.2%)	12 (32.4%)	1.25 [0.35–4.4]		0.85 [0.15–4.88]	
	Dominant						
	A/A	10 (30.3%)	16 (43.2%)	1	0.26176	1	0.08921
	A/G-G/G	23 (69.7%)	21 (56.8%)	0.57 [0.21–1.53]		0.32 [0.08–1.24]	
	Recessive						
	A/A-A/G	27 (81.8%)	25 (67.6%)	1	0.16954	1	0.42866
	G/G	6 (18.2%)	12 (32.4%)	2.16 [0.7–6.63]		1.86 [0.4–8.72]	
	Overdominant						
	A/A-G/G	16 (48.5%)	28 (75.7%)	1	0.01809	1	0.01735
	A/G	17 (51.5%)	9 (24.3%)	0.3 [0.11–0.83]		0.19 [0.04–0.84]	
	Log-Additive						
	0,1,2	33 (47.1%)	37 (52.9%)	1.02 [0.56–1.86]	0.94438	0.75 [0.32–1.74]	0.4939
rs2516839	Codominant						
	C/C	15 (50%)	6 (20%)	1	0.02099	1	0.02357
	C/T	9 (30%)	19 (63.3%)	5.28 [1.53–18.15]		9.58 [1.53–59.85]	
	T/T	6 (20%)	5 (16.7%)	2.08 [0.46–9.51]		5.08 [0.55–47.08]	
	Dominant						
	C/C	15 (50%)	6 (20%)	1	0.01367	1	0.0079
	C/T-T/T	15 (50%)	24 (80%)	4 [1.27–12.58]		8.13 [1.41–46.96]	
	Recessive						
	C/C-C/T	24 (80%)	25 (83.3%)	1	0.73851	1	0.84195
	T/T	6 (20%)	5 (16.7%)	0.8 [0.22–2.97]		1.19 [0.21–6.7]	
	Overdominant						
	C/C-T/T	21 (70%)	11 (36.7%)	1	0.00897	1	0.02085
	C/T	9 (30%)	19 (63.3%)	4.03 [1.37–11.84]		4.93 [1.19–20.48]	
	Log-Additive						
	0,1,2	30 (50%)	30 (50%)	1.72 [0.82–3.59]	0.14395	2.54 [0.91–7.06]	0.06087

## Data Availability

The data presented in this study are available on request from the corresponding author.

## Supplementary Materials

The following are available online at https://www.mdpi.com/article/10.3390/jcm14113850/s1, Table S1: Basic SNP Information—Chromosome, Position and Gene; Table S2: Results of population genetics based on the 1000 genomes project.
