# Supplementary material for: Genetic Variants in Preeclampsia During Pregnancy: A Hospital-Based Case–Control Study
Source: J Clin Med. 2025 May 30;14(11):3850. doi: 10.3390/jcm14113850 (PMC12156003; doi:10.3390/jcm14113850)
Supplement: Supplementary file 1 [file jcm-14-03850-s001.zip › jcm-3574926-supplementary.pdf]

Table S1. Basic SNP Information – Chromosome, Position and Gene.

Variation ID	Chromosome	Position	Gene
rs11206510	chr1	55030366	BSND*
rs599839	chr1	109279544	CELSR2*
rs2073658	chr1	161040972	USF1
rs2516839	chr1	161043331	USF1
rs2774279	chr1	161047766	ARHGAP30
rs6025	chr1	169549811	F5
rs5361	chr1	169731919	C1orf112
rs5361	chr1	169731919	SELE
rs1234313	chr1	173197108	TNFSF4
rs3850641	chr1	173206693	TNFSF4
rs1234315	chr1	173209324	TNFSF4*
rs17465637	chr1	222650187	MIA3
rs17672135	chr1	240282296	FMN2
rs501120	chr10	44258419	LINC00841*
rs1746048	chr10	44280376	AL137026.2*
rs1799963	chr11	46739505	F2
rs5443	chr12	6845711	GNB3
rs5443	chr12	6845711	CDCA3
rs3184504	chr12	111446804	SH2B3
rs3184504	chr12	111446804	ATXN2
rs3782886	chr12	111672685	BRAP
rs9536314	chr13	33054001	KL
rs2230500	chr14	61457521	PRKCH
rs1800588	chr15	58431476	ALDH1A2
rs1800588	chr15	58431476	LIPC
rs17228212	chr15	67166301	SMAD3
rs1051730	chr15	78601997	CHRNA3
rs183130	chr16	56957451	HERPUD1*
rs8055236	chr16	83178793	CDH13
rs5918	chr17	47283364	ITGB3
rs5918	chr17	47283364	AC068234.1
rs2229616	chr18	60372043	AC091576.1
rs2229616	chr18	60372043	MC4R
rs7250581	chr19	29573489	VSTM2B*
rs788016	chr2	197487569	HSPD1
rs2340690	chr2	197496162	HSPD1
rs6725887	chr2	202881162	WDR12
rs2943634	chr2	226203364	AC062015.1*
rs3843763	chr20	45919554	PLTP*
rs17576	chr20	46011586	MMP9
rs688034	chr22	26293669	SEZ6L
rs3803	chr3	128480537	GATA2
rs2713604	chr3	128481616	GATA2
rs2306374	chr3	138401110	MRAS
rs2200733	chr4	110789013	PITX2*
rs10033464	chr4	110799605	LINC01438*

rs1800787	chr4	154562863	AC107385.1*
rs383830	chr5	100613278	FAM174A*
rs2243250	chr5	132673462	TH2LCRR*
rs1042714	chr5	148826910	ADRB2
rs5370	chr6	12296022	EDN1
rs6922269	chr6	150931849	MTHFD1L
rs1800783	chr7	150992309	NOS3
rs1800779	chr7	150992855	NOS3
rs1799983	chr7	150999023	NOS3
rs268	chr8	19956018	LPL
rs326	chr8	19961928	LPL
rs10116277	chr9	22081398	CDKN2B-AS1
rs10757274	chr9	22096056	CDKN2B-AS1
rs2383206	chr9	22115027	CDKN2B-AS1
rs2383207	chr9	22115960	CDKN2B-AS1
rs1537375	chr9	22116072	CDKN2B-AS1
rs10757278	chr9	22124478	CDKN2B-AS1*
rs1333049	chr9	22125504	CDKN2B-AS1*
rs4986790	chr9	117713024	AL160272.2
rs4986790	chr9	117713024	TLR4
rs207044	chrX	39692049	MIR3937*

* - closest gene.

Table S2. Results of population genetics based on the 1000 genomes project.

Variation ID	REF Allele	ALT Allele	Minor Allele	AFR	AMR	EAS	EUR	SAS
rs11206510	T	C	C	0.1346	0.1167	0.0446	0.172	0.0337
rs599839	G	A	G	0.1785	0.7478	0.9345	0.7783	0.7382
rs2073658	C	T	T	0.0393	0.2651	0.1925	0.2942	0.1339
rs2516839	C	T	T	0.1846	0.4654	0.3333	0.6292	0.4611
rs2774279	C	T	T	0.1422	0.2003	0.1389	0.333	0.2975
rs6025	C	T	T		0.0101		0.0119	0.0112
rs5361	T	G	G	0.0257	0.0634	0.0119	0.0984	0.0879
rs1234313	A	G	A	0.8933	0.5735	0.3462	0.6859	0.5573
rs3850641	A	G	G	0.0499	0.1499	0.1786	0.168	0.2045
rs1234315	C	T	C	0.8366	0.5735	0.4216	0.4811	0.4387
rs17465637	A	C	A	0.1868	0.5144	0.6052	0.7376	0.5798
rs17672135	T	C	C	0.1044	0.0893	0.1488	0.1093	0.1554
rs501120	T	C	C	0.5023	0.2233	0.3423	0.1461	0.363
rs1746048	C	T	T	0.5446	0.2305	0.3105	0.1451	0.3528
rs1799963	G	A	A		0.0144		0.008	
rs3184504	T	C	T	0.9811	0.7464	0.997	0.5358	0.9315
rs3782886	T	C	C		0.0029	0.1746		
rs9536314	T	G	G	0.2005	0.0821		0.1938	0.137
rs2230500	G	A	A		0.0072	0.2688	0.008	0.0194
rs1800588	C	T	T	0.5598	0.4222	0.3998	0.2087	0.2955
rs17228212	T	C	C	0.0976	0.1326	0.001	0.2903	0.1227
rs1051730	G	A	A	0.087	0.2161	0.0268	0.3688	0.183
rs183130	C	T	T	0.2655	0.3026	0.1746	0.2932	0.319
rs8055236	G	T	T	0.5772	0.1239	0.131	0.1819	0.1513
rs5918	T	C	C	0.0915	0.1023	0.0089	0.1322	0.1135
rs2229616	C	T	T	0.0197	0.0086	0.0179	0.007	0.0245
rs7250581	A	G	A	0.9955	0.9294	1	0.837	0.9785
rs788016	G	A	A	0.3124	0.4092	0.2431	0.5139	0.5736
rs2340690	A	G	G	0.053	0.1715	0.2282	0.2117	0.0951
rs6725887	T	C	C	0.0174	0.0735	0.0159	0.1302	0.0143
rs2943634	A	C	A	0.385	0.7507	0.9157	0.6521	0.8037
rs2943634	A	G	A		0.0029		0.007	0.002
rs3843763	C	T	T	0.3933	0.1974	0.372	0.2525	0.2945
rs17576	A	G	G	0.3442	0.2277	0.7391	0.3807	0.5521
rs688034	C	T	T	0.0688	0.17	0.002	0.3082	0.0798
rs3803	G	A	A	0.1634	0.134	0.0556	0.1978	0.0613
rs2713604	T	C	T	0.7761	0.6945	0.6796	0.6978	0.5736
rs2306374	T	C	C	0.0681	0.0634	0.0258	0.1451	0.091
rs2200733	C	T	T	0.2315	0.2767	0.4514	0.1551	0.1309
rs10033464	T	G	T	0.8086	0.8487	0.745	0.9026	0.8006
rs1800787	C	T	T	0.0908	0.1542	0.2222	0.2167	0.1401
rs383830	A	T	A	0.6225	0.8573	0.8482	0.8101	0.8384
rs2243250	C	T	T	0.73	0.366	0.7788	0.168	0.184
rs1042714	G	C	G	0.8638	0.7579	0.9266	0.5905	0.8067
rs1042714	G	T	G					
rs5370	G	T	T	0.1619	0.147	0.2857	0.2127	0.4305

rs6922269	G	A	A	0.6104	0.3256	0.0337	0.2654	0.2883
rs1800783	A	T	A	0.5318	0.7104	0.881	0.5626	0.7423
rs1800779	G	A	G	0.8608	0.745	0.881	0.5686	0.7505
rs1799983	T	G	T	0.9297	0.7853	0.87	0.6561	0.8323
rs268	A	G	G	0.0008	0.0115		0.0139	0.0031
rs326	A	G	G	0.5862	0.2867	0.2341	0.327	0.2157
rs10116277	G	T	G	0.9682	0.4885	0.7044	0.4851	0.5838
rs10757274	A	G	G	0.1755	0.4179	0.5288	0.492	0.4836
rs2383206	A	G	G	0.413	0.5	0.5258	0.507	0.5204
rs2383207	A	G	A	0.9667	0.5461	0.6925	0.508	0.5982
rs1537375	T	C	T	0.7254	0.5101	0.6925	0.502	0.5716
rs10757278	A	G	G	0.1589	0.4553	0.5437	0.4742	0.5041
rs1333049	G	C	C	0.2133	0.4553	0.5367	0.4722	0.4908
rs4986790	A	G	G	0.0711	0.0375		0.0567	0.1258
rs207044	C	T	T	0.4716	0.3836	0.5275	0.4347	0.2298
